# Composition-dependent structure of polycrystalline magnetron-sputtered V–Al–C–N hard coatings studied by XRD, XPS, XANES and EXAFS

**DOI:** 10.1107/S0021889813014477

**Published:** 2013-07-18

**Authors:** Bärbel Krause, Susan Darma, Marthe Kaufholz, Stefan Mangold, Stephen Doyle, Sven Ulrich, Harald Leiste, Michael Stüber, Tilo Baumbach

**Affiliations:** a Institut für Photonenforschung und Synchrotronstrahlung (IPS), Karlsruher Institut für Technologie, Karlsruhe, Germany; b ANKA Synchrotronstrahlungsquelle, Karlsruher Institut für Technologie, Karlsruhe, Germany; c Institut für Angewandte Materialien, Angewandte Werkstoffphysik, Karlsruher Institut für Technologie, Karlsruhe, Germany

**Keywords:** sputter deposition, hard coatings, metastable phases, nitrides, carbides, amorphous carbon, X-ray photoelectron spectroscopy (XPS), X-ray absorption near-edge spectroscopy (XANES), extended X-ray absorption fine-structure spectroscopy (EXAFS)

## Abstract

V–Al–C–N hard coatings with high carbon content were deposited by reactive radio-frequency magnetron sputtering using an experimental combinatorial approach, deposition from a segmented sputter target. The composition-dependent coexisting phases within the coating were analysed using the complementary methods of X-ray diffraction (XRD), X-ray photoelectron spectroscopy (XPS), X-ray absorption near-edge spectroscopy (XANES) and extended X-ray absorption fine-structure spectroscopy (EXAFS). For the analysis of the X-ray absorption near-edge spectra, a new approach for evaluation of the pre-edge peak was developed, taking into account the self-absorption effects in thin films. Within the studied composition range, a mixed face-centred cubic (V,Al)(C,N) phase coexisting with a C–C-containing phase was observed. No indication of hexagonal (V,Al)(N,C) was found. The example of V–Al–C–N demonstrates how important a combination of complementary methods is for the detection of coexisting phases in complex multi-element coatings.

## Abstract 2

V–Al–C–N hard coatings with high carbon content were deposited by reactive radio-frequency magnetron sputtering from a segmented sputter target. The composition-dependent coexisting phases were analysed using the complementary methods of X-ray diffraction (XRD), X-ray photoelectron spectroscopy (XPS), X-ray absorption near-edge spectroscopy (XANES) and extended X-ray absorption fine-structure spectroscopy (EXAFS).

## 1. Introduction   

Complex hard coating materials based on transition element (TM) nitrides and carbides, such as CrAlN, TiAlN and TiCN, are nowadays commercially available and widely used for industrial applications. Besides the high hardness, the desired coating properties usually also include temperature stability, wear and corrosion resistance. These properties are closely related to the chemical composition and the microstructure of the coatings.

Multi-element coatings provide many options for tuning their properties (Holleck, 1990 ▶; Mayrhofer *et al.*, 2006 ▶). One of them is the controlled formation of coexisting crystalline and amorphous phases. For sputter-deposited TM–Al–N coatings, a very interesting behaviour was observed. From thermodynamic equilibrium considerations, the following scenario is expected: below a critical AlN concentration *c*_1_, a single mixed cubic phase forms, followed by coexisting cubic and hexagonal mixed phases in the concentration range *c*_1_ < *c* < *c*_2_. Above *c*_2_, only a single mixed hexagonal phase exists. This scenario has been confirmed experimentally, *e.g.* by Ikeda & Satoh (1991 ▶) and Holec *et al.* (2011 ▶). However, non-equilibrium growth conditions can also result in a second scenario for the as-deposited coating: a single metastable mixed phase can form between *c*_1_ and *c*_2_, which is cubic for *c*_1_ < *c* < *c*_t_ and hexagonal for *c*_t_ < *c* < *c*_2_ (*c*_t_ is the theoretically predicted solubility limit). Typical experimental values are in the ranges 0.4–0.6 for *c*_1_ and 0.6–0.8 for *c*_2_ (Zhou *et al.*, 1999 ▶; Tuilier *et al.*, 2007 ▶; Holec *et al.*, 2011 ▶). The value of *c*_t_ for AlN in the metastable cubic mixed phase is around 0.7 (Mayrhofer *et al.*, 2007 ▶; Holec *et al.*, 2011 ▶).

The crystalline phases are very important for the properties of the coating, as was shown for the system Ti–Al–N. For Ti–Al–N deposited in the thermodynamic equilibrium scenario, the highest hardness was demonstrated for the cubic phase with concentrations close to *c*_1_ (Zhou *et al.*, 1999 ▶). In contrast, for metastable Ti–Al–N the highest hardness was found for the cubic structure with *c* close to *c*_t_ (PalDey & Deevi, 2003 ▶). For the metastable mixed cubic phase of Ti–Al–N, spinodal decomposition into cubic nanodomains of TiN and AlN can occur after deposition, leading to age hardening of the coatings (Mayrhofer *et al.*, 2003 ▶; Alling *et al.*, 2007 ▶).

Not only the hardness but also the friction coefficient of a multi-element coating is influenced strongly by the coexisting phases. For Ti–C, it was shown that, at sufficiently high carbon content, two separate phases form during deposition, cubic TiC_1−*x*_ and amorphous carbon. An amorphous carbon matrix or grain-boundary phase can contribute to the optimized friction and wear behaviour of such materials, resulting in low-friction or even solid lubricant materials (Zehnder & Patscheider, 2000 ▶; Stüber *et al.*, 2002 ▶). A similar amorphous carbon phase was also observed for Ti–Al–C–N (Shieh & Hon, 2002 ▶; Lackner *et al.*, 2004 ▶; Stueber *et al.*, 2005 ▶; Nose *et al.*, 2010 ▶).

Both theoretical considerations and initial experimental studies suggest that V–Al–C–N might be a very promising candidate for hard coating applications (Kolozsvári *et al.*, 2009 ▶; Ziebert *et al.*, 2009 ▶; Rovere *et al.*, 2010 ▶; Stüber *et al.*, 2011 ▶). The material system is expected to combine the properties of the metallic hard material VC_1−*x*_ and the covalent hard material AlN. In analogy with Ti–Al–C–N, the desired crystalline phase is a metastable face-centred cubic (f.c.c.) mixed (V,Al)(C,N) phase. A large AlN content might increase the hardness. The formation of a V_2_O_5_ surface oxide layer and a coexisting amorphous carbon phase at sufficiently high carbon contents is expected to reduce the friction coefficient.

In this paper, a systematic study of the composition-dependent phases formed by V–Al–C–N will be presented. In order to determine the optimum composition and deposition conditions of these complex materials, many samples are required. One powerful tool for reducing the number of sample preparations is based on the so-called combinatorial approach for thin-film deposition: many different thin-film samples of varying compositions can be realized simultaneously, exploiting the deposition gradient resulting from codeposition of several materials (Holleck & Lahres, 1991 ▶; Mitterer *et al.*, 1998 ▶; Wang *et al.*, 2002 ▶; Ludwig *et al.*, 2008 ▶; Stüber *et al.*, 2008 ▶). The coatings studied here were deposited simultaneously by reactive radio-frequency (RF) magnetron sputtering from a segmented VC/AlN target, as described by Ziebert *et al.* (2009 ▶).

For multi-element coatings, the determination of the crystalline phases can be very challenging. In many cases, this task can only be performed using a combination of complementary experimental methods. In this paper, we will present the results of a combined X-ray diffraction (XRD), extended X-ray absorption fine-structure spectroscopy (EXAFS), X-ray absorption near-edge structure spectroscopy (XANES) and X-ray photoelectron spectroscopy (XPS) study of V–Al–C–N. For the interpretation of these measurements, the thickness and chemical composition of the samples were determined independently. In the following, the strong points of each method for the understanding of our new V–Al–C–N material system are summarized.

XRD gives information about the long-range order of the crystalline phases. Different crystal structures, such as hexagonal and cubic mixed (V,Al)(C,N), can be distinguished by their characteristic Bragg peak positions. However, owing to the superposition of texture, size and strain effects, it can be very difficult to distinguish between different phases with the same crystal structure and similar lattice parameters but different chemical compositions. In the case of V–Al–C–N, these are, for instance, the possible cubic phases VC_1−*x*_ and (V,Al)(C,N).

X-ray absorption spectroscopy (XAS) measurements in terms of EXAFS and XANES measurements extend the range of detectable phases to short-range-ordered and amorphous materials. They give information about the local order around the absorbing atom and the bond length to neighbouring atoms. This allows, for instance, the detection of small hexagonal clusters within a cubic matrix, which might be invisible in the XRD measurement.

The additional information about the chemical bond provided by XPS narrows the range of possible coexisting phases. In contrast with XRD and XAS, the information depth is limited to the topmost few nanometres of the thin film. One advantage of XPS is that it can detect all elements in the coating, while it is difficult to distinguish C and N by XRD and EXAFS.

The aim of this paper is to analyse the coexisting amorphous and crystalline phases in V–Al–C–N. For this, a new approach to evaluation of the XANES pre-edge peak was developed, taking into account the self-absorption effects in thin films. Both the experimental and methodological results will be presented.

The paper is structured as follows: the details of the experiment and the data analysis are summarized in §2. The thickness and composition of the samples and the results of the XRD, XAFS and XPS measurements are presented and discussed in §3, with a summary in §4.

## 2. Experimental   

### 2.1. Sample description   

**Figure 1:**
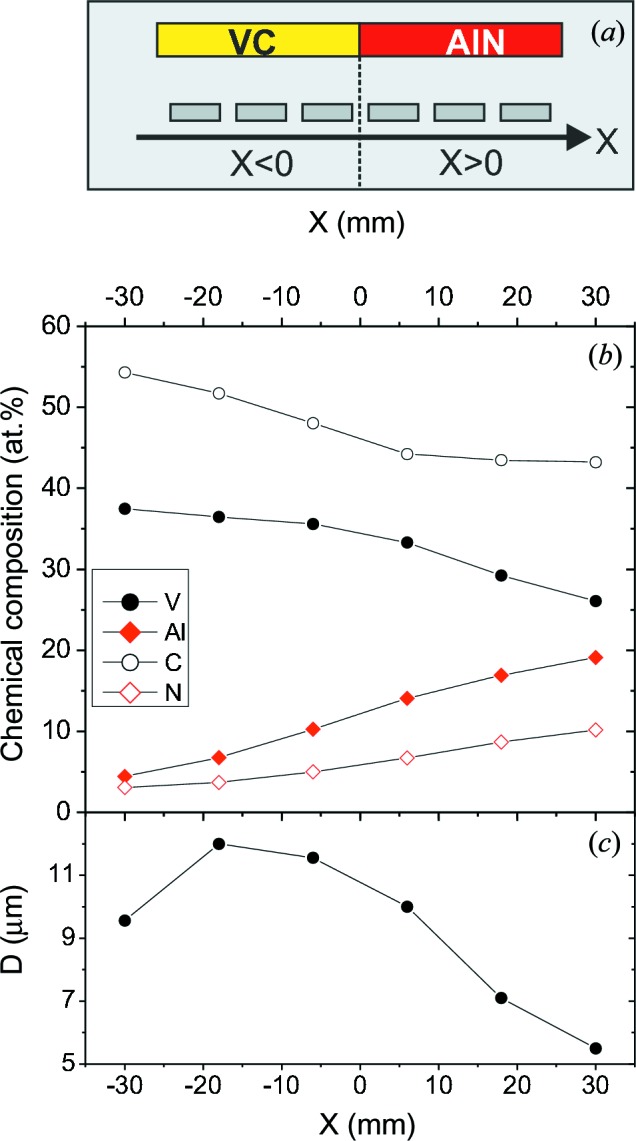
(*a*) Schematic of the experimental setup for thin-film deposition, using a segmented VC/AlN target. (*b*) Chemical compositon of V–Al–C–N thin films at different positions *X* below the target. (*c*) Coating thickness at different positions *X*.

The V–Al–C–N thin films discussed in this paper were deposited simultaneously by reactive RF magnetron sputtering from a segmented VC/AlN target, as shown schematically in Fig. 1 ▶(*a*). This experimental combinatorial approach results in a composition gradient along the *X* axis of the deposited V–Al–C–N thin films, which is accompanied by a local thickness variation. The chemical composition as measured by electron-probe microanalysis (EPMA) with a Camebax Microbeam apparatus is shown in Fig. 1 ▶(*b*). The values were averaged over three to ten measurements with a spot size of 5 µm. The coating thickness as measured with a Calo tester (CSEM SA, Switzerland) close to the centre of the samples is shown in Fig. 1 ▶(*c*).

The details of the thin-film deposition are given by Ziebert *et al.* (2009 ▶). In the following, the main process parameters will be summarized. Six cemented carbide substrates (consisting mainly of WC) with a surface area of 12 × 12 mm and a thickness of 4.5 mm were placed along the gradient direction. The sample position *X* is negative below the VC segment and positive below the AlN target segment. During deposition, the total gas flow of the Ar sputter gas with the addition of 2 vol.% of the reactive gas CH_4_ was 60 standard cubic centimetres per minute, corresponding to a total pressure of 0.6 Pa. The power density was 11.3 W cm^−2^ and no substrate bias was applied. The substrate temperature was 423 K and the deposition time was 4 h. A second set of V–Al–C–N thin films was deposited on Si(100) substrates, with similar deposition parameters but a reduced deposition time of 75 min. As a result of the different deposition time, the samples deposited on WC are about three times thicker than those deposited on Si, thus allowing the study of thickness effects on the measurements. It must be noted that, because of the different substrates and coating thicknesses, the microstructure of the two coatings is not identical.

Additionally, a VC_1−*x*_ coating and a VN_1−*x*_ coating were deposited on cemented carbide using a standard reactive RF magnetron sputtering process with a vanadium target. The VC_1−*x*_ coating, with a thickness *D* = 4.1 µm, is understoichiometric with *x* = 0.2, while the VN_1−*x*_ coating, with *D* = 5.9 µm, is understoichiometric with *x* = 0.15. The reference coatings are nanocrystalline with coexisting (111) and (200) textures. A second VC_1−*x*_ reference coating, with *D* = 40 nm, was deposited by DC magnetron sputtering from a nominally stoichiometric VC target. The exact stoichiometry of the coating is unknown. In the following, the reference coatings will be referred to as the VC, VN and 40 nm VC reference samples.

Since the reference samples were deposited under various growth conditions, they are only used as reference for certain features related to the crystalline unit cell and the self-absorption effect in the XANES measurements.

### 2.2. X-ray diffraction   

XRD measurements were performed at the powder diffraction beamline PDIFF of the synchrotron facility Ångströmquelle Karlsruhe (ANKA, Karlsruhe, Germany). For the measurements, an Oxford Diffraction Kappa diffractometer was used. The X-ray beam, with an energy *E* = 9.5 keV corresponding to a wavelength λ = 1.305 Å, was focused to a size of about 2 × 2 mm on the sample position. The scattered X-ray intensity was collected with a scintillation detector with an Si(111) analyser for better energy resolution.

All samples were characterized by XRD radial scans, also called ω/2θ scans, where the sample angle ω is varied by steps of Δω and the detector angle 2θ is varied by steps of Δ2θ = 2Δω. The scans covered the range *Q* = 2.25–6.25 Å^−1^, where the scattering vector magnitude *Q* in reciprocal space is defined as
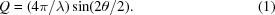
As verified by a preliminary analysis of the pole figure measurements of the 111 and 200 reflections, the V–Al–C–N samples are only weakly textured, *i.e.* the intensity of all Bragg reflections is distributed continuously along the respective powder ring but has maxima at certain positions (a detailed analysis will be published separately). Therefore, radial scans are sufficient for the determination of the crystalline phase. For the VC reference coating, the radial scan was performed along the surface normal, while for the V–Al–C–N samples it was measured in the direction of the preferential (111) texture. For selected V–Al–C–N samples, strain maps consisting of several radial scans measured at different tilt angles ψ with respect to the surface normal were recorded. From these measurements, the lateral compression of the (111) texture was determined.

### 2.3. X-ray photoelectron spectroscopy   

XPS measurements were performed in the ANKA UHV analysis laboratory, using a Phoibos 150 analyser and an unmonochromated XR-50 Mg *K*α X-ray source from SPECS. The angle between the analyser and the X-ray source was 45° and the electrons emitted from a sample area with a diameter of about 2 mm were detected along the surface normal of the sample. The energy scale was verified with a precision of ±0.05 eV using the Cu 2*p*_1/2_ XPS peak at 932.62 eV and the Cu *L*_3_ VV Auger peak at 334.90 eV (Briggs & Grant, 2003 ▶). The base pressure of the XPS chamber was 1 × 10^−8^ Pa. Since the samples were exposed to ambient conditions prior to the XPS measurements, they were sputter cleaned with 3 keV Ar ions for about 1 h, using a scanning ion gun (IQE 12/38, SPECS) with a spot size of about 0.7 mm, an ion current of 3 µA and a scan range of 6 × 6 mm. XPS measurements were performed before and after the Ar sputtering. No beam-induced changes in the spectra were detected.

### 2.4. X-ray absorption near-edge spectroscopy and extended X-ray absorption fine-structure spectroscopy   

EXAFS measurements at the vanadium *K* edge at *E* = 5.465 keV were carried out on the XAS beamline at ANKA. For the energy calibration of the beamline with a precision of ±0.2 eV, a V metal foil was used. The samples were mounted on a sample holder with motorized *xyz* translation and rotation for the incident angle. The beam size of 1 × 1 mm at the sample position was defined by slits. Owing to the substantial thickness of the substrate, the measurements were performed in fluorescence geometry. The incident beam was monitored with an ionization chamber (IC Spec, manufactured by FMB Oxford) filled with an He/N_2_ mixture optimized for 15% absorption at the V edge. The fluorescence intensity of V *K*α_1_ with *E* = 4.953 keV was recorded with a five-element Ge detector (Canberra). Both incident angle ϕ and detector angle θ were typically 45° with respect to the sample surface. For selected samples, additional measurements at different incident angles were performed in order to study the influence of self-absorption. For these measurements, the detector angle was θ = 90° − ϕ, *i.e.* the angle between the incident beam and the detector was always 90°.

Data evaluation was performed using the package *IFEFFIT* (Newville, 2001 ▶) (*FEFF* code, Version *FEFF6*), using the software *Athena* (Ravel & Newville, 2005 ▶) for background correction and the program *Artemis* (Ravel & Newville, 2005 ▶) for the fit, with the *k* weights 1 and 3 in the *R* space in the range 1–3 Å. The Fourier transform was performed in the *k* range 2–11.5 Å^−1^.

The V–Al–C–N coatings studied here, with thicknesses in the micrometre range, are strongly absorbing samples, *i.e.* the measured absorption coefficient is no longer proportional to the concentration of the absorbing atoms. The measured V *K*-edge spectrum of a thin layer of thickness *D* is related to the absorption coefficient μ_V_(*E*) of the V atoms *via*

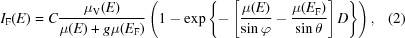
where μ(*E*) and μ(*E*_F_) are the total absorption coefficients of the coating at the measurement energy *E* and at the energy of the fluorescence radiation *E*_F_, *g* is the geometry factor with *g* = sinϕ/sinθ, and *C* is a proportionality factor which varies so slowly with *E* that it can be treated as energy independent (Tröger *et al.*, 1992 ▶). Several solutions exist for calculating μ_V_(*E*) from this nonlinear equation. The algorithm of Booth & Bridges (2005 ▶), implemented in *Athena*, is valid for the EXAFS region of coatings of finite thickness. All EXAFS data were absorption corrected with the Booth algorithm, using the measured chemical composition and coating thickness. However, for the XANES region only the correction algorithm *FLUO* exists (http://www.aps.anl.gov/xfd/people/haskel/fluo.html), which is limited to samples with a large coating thickness compared with the absorption length. In our case, this assumption is not fulfilled.

Since our coatings have a high V and C content, it is instructive to compare the measured spectra with spectra from thin VC coatings of similar thickness. The 40 nm VC reference sample is so thin that the self-absorption effect can be neglected. From the measured spectrum, the absorption coefficient μ_V_(*E*) was determined and inserted into equation (2). The expected *I*_F_(*E*) were calculated for different coating thicknesses *D*. The resulting spectrum (referred to as the calculated XANES spectrum) was normalized using the program *Athena*.

## 3. Results   

### 3.1. Chemical composition and thickness variation   

Fig. 1 ▶(*b*) shows the chemical composition measured by EPMA. The concentrations of the respective atom types, V, Al, C and N, are given in atomic percent. For all samples, the main constituents are carbon, with a concentration of ∼55–45 at.%, vanadium, with a concentration of 38–26 at.%, and aluminium, with a concentration of ∼5–20 at.%. The constituent with the lowest concentration is nitrogen, at ∼3–10 at.%. Both the argon content (0.5 at.%) and the oxygen content (1–2 at.%) are negligible. However, the absolute values of the oxygen content are not reliable, since the EPMA signals for O and V overlap strongly.

As a result of the composition gradient obtained during deposition, the concentration ratio Al/(V + Al) varies from about 0.1 for *X* = −30 mm to 0.4 for *X* = 30 mm. For all samples the nitrogen content is always less than the aluminium content and the carbon content is larger than the vanadium content, *i.e.* compared with the target composition there is a nitrogen deficit and a carbon excess. The metal:nonmetal ratio (V + Al)/(C + N) is always less than 1. The coating thickness shown in Fig. 1 ▶(*c*) has a maximum of about 12 µm at *X* = −18 mm and decreases in both directions, with a minimum of 5.5 µm at *X* = 30 mm.

Each measurement method used here averages the signal over a certain spot size. Assuming, for instance, a spot size of 1 mm, the observed maximum variation in local thickness within the spot is ±100 nm and the maximum concentration variation is ±0.25 at.%. The spot sizes of the EXAFS and XRD measurements were chosen after verifying experimentally (using different spot sizes) that the signal was not visibly influenced by the gradient.

### 3.2. X-ray diffraction   

**Figure 2:**
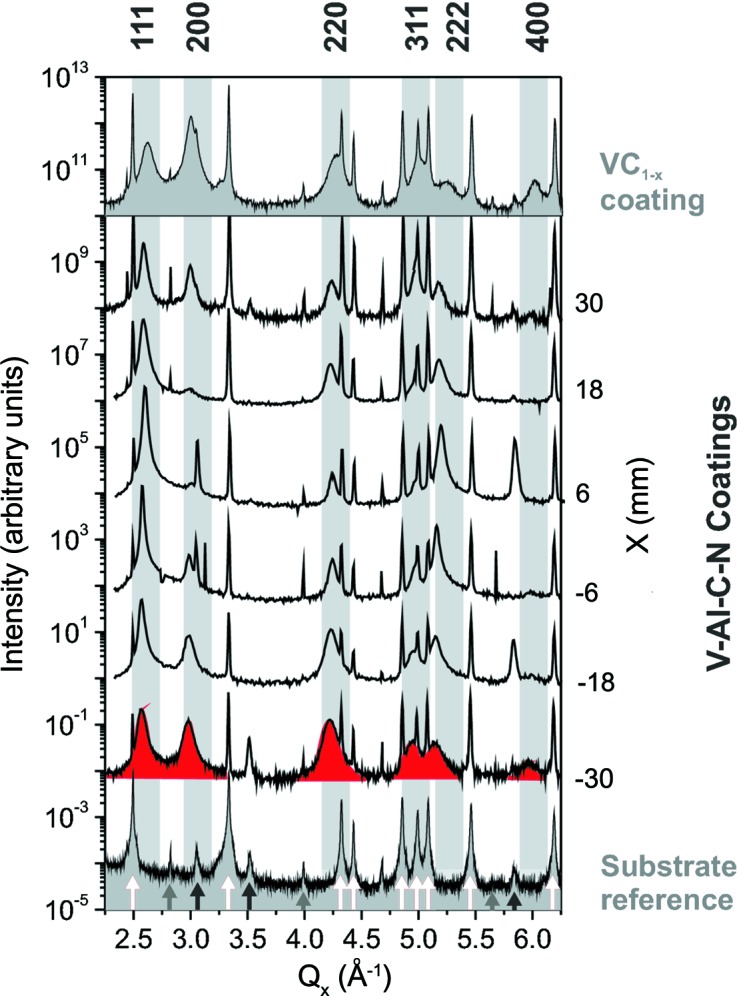
Radial scans of the substrate, the V–Al–C–N coatings and the VC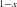 reference coating. For the sample at 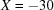
 mm, the broad coating peaks are shaded in dark grey (red in the electronic version of the journal). The allowed peaks for the f.c.c. NaCl structure are indicated.

Fig. 2 ▶ shows radial scans of the V–Al–C–N coatings deposited on cemented carbide substrates. The position *X* below the target is indicated on the right-hand side. For comparison, radial scans of a substrate (bottom spectrum) and a VC_1−*x*_ coating (top spectrum) are presented. The reference measurements are shaded in mid-grey.

The strongest peaks of the substrate reference are indicated by white arrows, and they can be attributed to the δ-WC phase (Kurlov & Gusev, 2006 ▶). The weaker peaks are mainly related to hexagonal close-packed (h.c.p.) Co (black arrows) and body-centred cubic W (grey arrows). Substrate peaks are observed in the radial scans of all the V–Al–C–N coatings and in the VC_1−*x*_ reference. However, their intensity changes and some peaks are only weakly visible or completely missing. The fluctuation in peak intensity is related to a certain lateral inhomogeneity of the substrate texture. The total scattering signal of the substrate is reduced by the absorption of the coating, which varies with the coating thickness, *i.e.* with the *X* position for the V–Al–C–N coatings.

In comparison with the substrate peaks, the coating peaks are very broad. The VC_1−*x*_ reference coating shows the typical rock-salt structure expected for both VC_1−*x*_ and VN. The allowed peaks for the f.c.c. rock-salt structure are indicated by grey bars superposed on the spectra. Similarly to the VC_1−*x*_ reference sample, for all V–Al–C–N coatings broad reflections corresponding to the f.c.c. rock salt structure are observed. For the sample at *X* = −30 mm, the broad coating peaks are shaded dark grey (red in the electonic version of the journal). The spectra show no features related to other crystalline or amorphous phases.

For all V–Al–C–N coatings, the most intense and narrowest reflection is the 111 peak, which is related to the preferential (111) fibre texture observed for all the coatings. However, other weaker textures such as (220) and (200) coexist. Williamson–Hall analysis (see *e.g.* Birkholz, 2006 ▶) of the peak widths showed that the minimum value for the mean crystallite size is about 10 ± 3 nm.

**Figure 3:**
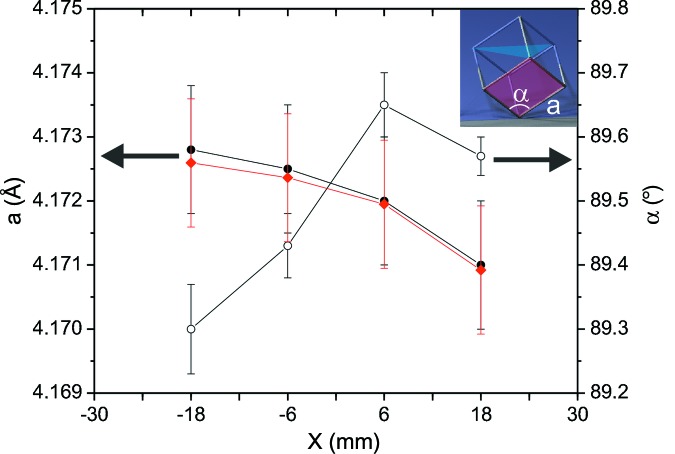
Unit-cell parameters 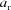 (black dots, left axis) and α (black open circles, right axis) of the laterally compressed (111) crystallites of V–Al–C–N thin films deposited at different positions *X*. The inset shows a schematic of the rhombohedrally distorted unit cell. The shaded diamonds correspond to the calculated lattice parameter *a* for the relaxed cubic unit cell.

For the samples with −18 ≤ *X* ≤ 18 mm, the radial positions of the 111, 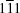, 200 and 220 reflections of the dominant (111) fibre texture were determined from the strain maps. It was found that the [111]-oriented crystallites are distorted owing to the lateral compression of the coating. Fig. 3 ▶ shows the angle α (open circles) and lattice parameter *a*_r_ (black filled circles) of the rhombohedral unit cell resulting from this lateral compression. From this, the relaxed lattice parameter *a* of the cubic unit cell (diamonds) was calculated, assuming that the unit-cell volume does not change as a result of the slight lateral compression. The lattice parameter decreases with increasing *X*, *i.e.* with increasing AlN content. For a mixed phase this is expected from Vegard’s law, since cubic AlN has a smaller lattice parameter than cubic VC. However, a detailed comparison with the expected lattice parameters is difficult, since the reference values for both VC_1−*x*_ and cubic AlN are widely scattered. VC_1−*x*_ occurs over a wide concentration range with different lattice parameters, and in general the exact composition of the cubic phase is not known owing to possible coexistence with a carbon phase (Lipatnikov, 2005 ▶). The typical range of the reference values in the Inorganic Crystal Structure Database (ICSD, 2013 ▶) is 4.16–4.17 Å. Theoretical values for the lattice parameter of AlN are in the range 4.03–4.07 Å (Saib & Bouarissa, 2005 ▶; Zhang *et al.*, 2007 ▶; Wang *et al.*, 2010 ▶). Experimental values deviate somewhat, since the metastable cubic AlN is often stabilized in strained thin films and multilayers (Setoyama *et al.*, 1996 ▶; Kim *et al.*, 2001 ▶; Zhu *et al.*, 2008 ▶).

### 3.3. X-ray photoelectron spectroscopy   

**Figure 4:**
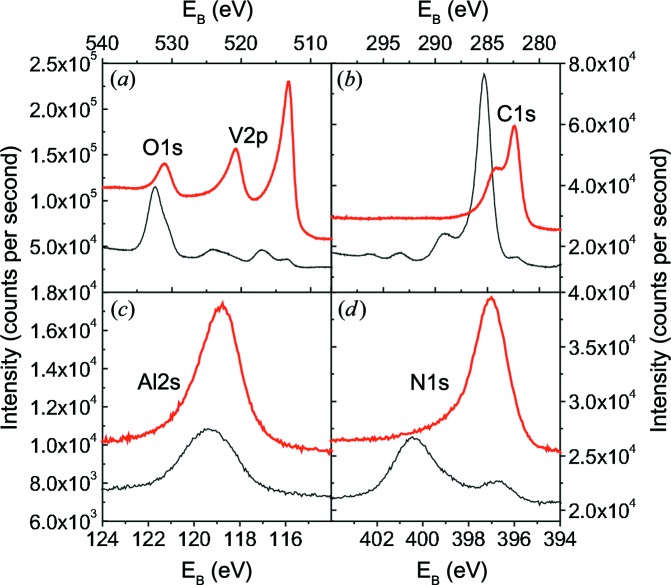
XPS spectra of the sample at 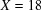
 mm before (lower spectrum, thin black line) and after (upper spectrum, thick line) the Ar sputter cleaning: (*a*) O1*s* and V2*p*, (*b*) C1*s*, (*c*) Al2*s*, and (*d*) N1s. For better comparison, the spectra are vertically shifted.

**Figure 5:**
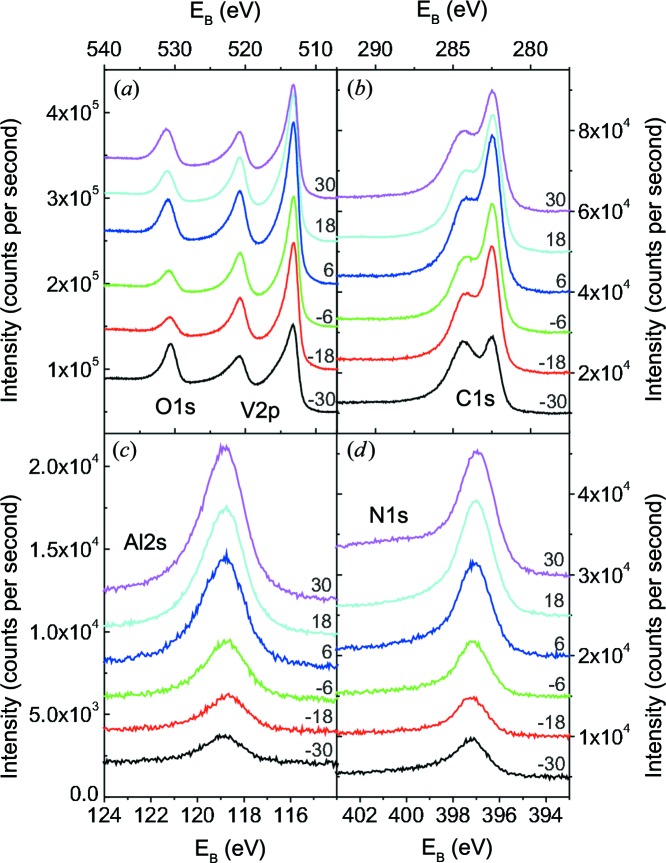
Comparison of the XPS specta measured after Ar^+^ ion bombardment at different sample positions *X*: (*a*) O1*s* and V2*p*, (*b*) C1*s*, (*c*) Al2*s*, and (*d*) N1*s*. For better comparison, the spectra are vertically shifted. The positions *X* in millimetres are indicated.

XPS measurements were performed for all samples deposited on cemented carbide substrates. As an example, Fig. 4 ▶ shows XPS spectra for the sample at *X* = 18 mm before (thin black line) and after (thick line) Ar^+^ ion bombardment. The XPS specta measured after Ar^+^ ion bombardment at different sample positions *X* are summarized in Fig. 5 ▶.

The XPS spectrum covering the O1*s* and the V2*p* peaks before sputtering (Fig. 4 ▶ *a*) shows a V2*p*_3/2_ peak at 517.10 eV and a V2*p*_1/2_ peak at 524.15 eV. These positions are characteristic for V—O bonds in V_2_O_5_ (Choi, 1999 ▶; Demeter *et al.*, 2000 ▶; Silversmit *et al.*, 2004 ▶). The maximum position of 532.45 eV for O1*s* is dominated by adsorbates such as H_2_O. After sputter cleaning, the O1*s* peak is observed at 531.15  eV, *i.e.* close to the shoulder of O1*s* already visible before Ar sputtering. The peak position is in between the expected O1*s* peak of V_2_O_5_ at about 530.0 eV (Mendialdua *et al.*, 1995 ▶; Guimond *et al.*, 2008 ▶; Zou *et al.*, 2009 ▶) and the reported O1*s* positions of Al_2_O_3_, which are typically in the range 531–533 eV (Hinnen *et al.*, 1994 ▶; van den Brand *et al.*, 2004 ▶; Snijders *et al.*, 2005 ▶). For the V–Al–C–N samples discussed here, the O1*s* and V2*p* spectra are similar (see Fig. 5 ▶ *a*). The O1*s* peak measured after Ar sputtering shifts from 530.6 to 531.2 eV with increasing *X*, *i.e.* with increasing Al content. All other XPS peaks remain unchanged to within ±0.1 eV. The O1*s* peak shift can be explained if we assume that the O1*s* peak is a superposition of the signal from Al—O and V—O bonds. With increasing Al content, the Al—O contribution is expected to increase, leading to a peak shift to higher binding energies.

After sputter cleaning, the V2*p*_3/2_ and V2*p*_1/2_ maxima are shifted to 513.25 and 520.85 eV, respectively, consistent with the reported values for both VC_1−*x*_ (Choi, 1999 ▶; Liao *et al.*, 2005 ▶) and VN (Sanjinés *et al.*, 1998 ▶; Liao *et al.*, 2004 ▶; Glaser *et al.*, 2007 ▶). The peak at about 513 eV is already observed before sputter cleaning, but with a much lower intensity. Since the XPS signal comes mainly from the topmost 2–3 nm of the thin film, this indicates that the oxide layer is restricted to the topmost layers of the coating.

Fig. 4 ▶(*b*) shows the C1*s* spectrum before and after Ar^+^ bombardment. Before sputter cleaning, the most intense C1*s* peak is found at 285.35 eV. After sputter cleaning, a strong peak at 282.45 eV, overlapping with a weaker peak at 284.15 eV, is observed. The strong peak, which is weakly visible even before sputter-cleaning, can be attributed to a carbide. The relative intensity of the two peaks seems to depend on *X*. This might be due to the different amounts of the two phases, but the microstructure and Ar treatment of the sample might also play a role. For both the V—C bond (Frantz & Didziulis, 1998 ▶; Choi, 1999 ▶; Liao *et al.*, 2005 ▶) and the Al—C bond (Hinnen *et al.*, 1994 ▶; Jiang *et al.*, 2002 ▶), C1*s* binding energies of about 282.5 eV are found in the literature. The weak peak originates from a C—C bond. Similar C1*s* spectra have already been reported for TiC/α-C nanocomposites (Stüber *et al.*, 2002 ▶; Magnuson *et al.*, 2009 ▶; Mel *et al.*, 2010 ▶). They might be related to the formation of carbon inclusions or a carbon grain-boundary phase in the V–Al–C–N concentration range studied here.

Most references for XPS measurements of Al refer to the Al2*p* spectrum. However, in our case Al2*p* overlaps partially with V3*s*. Therefore, Fig. 4 ▶(*c*) shows the Al2*s* peak, which shifts from 119.35 eV before to 118.8 eV after Ar^+^ bombardment. The observed peak position before sputter cleaning is close to the expected peak position of 119.41 eV for Al2*s* in Al_2_O_3_ (Unter *et al.*, 2000 ▶). We assume that, before cleaning, the peak is dominated by the Al—O bond. Since the Al—C and Al—N bonds are expected to be very similar, both might contribute to Al2*s* after sputter cleaning.

The N1*s* peak is shown in Fig. 4 ▶(*d*). Before Ar ion bombardment, a strong peak at 400.45 eV and a weak peak at 397.0 eV are observed. The second peak increases in intensity after sputter cleaning, while the first peak vanishes. For both AlN and VN, values of around 397 eV are reported (Liao *et al.*, 2004 ▶; Glaser *et al.*, 2007 ▶; Sanjinés *et al.*, 1998 ▶; Zhou *et al.*, 1999 ▶; Laidani *et al.*, 1999 ▶). Therefore, the peak at 397.0 eV is attributed to a nitride. The peak at 400.45 eV is related to surface adsorbates.

The intensity of Al2*s* and N1*s* increases significantly with increasing sample position *X*, as shown in Figs. 5 ▶(*c*) and 5 ▶(*d*). This is expected, since both the Al and the N content increase in this range by a factor of three to four (see Fig. 1 ▶). Since the relative change in V and C content is much less in the same range, the effect of the concentration on the peak intensity is less visible for V2*p* and C1*s*.

To summarize the XPS results: before sputter cleaning, the signal is dominated by adsorbates, V_2_O_5_ and Al_2_O_3_. Additionally, weak nitride and carbide signals are observed, indicating that the oxide signal comes mainly from the topmost 2–3 nm close to the sample surface. After sputter cleaning, the spectra are dominated by V—(C,N), Al—(C,N) and C—C. It is not possible to distinguish between the respective nitrides and carbides. A weak oxide signal is still visible. The O1*s* peak, which shifts with increasing Al content, indicates that both V—O and Al—O are present even after the Ar^+^ ion bombardment.

### 3.4. X-ray absorption near-edge structure   

In the following, the results of the XANES measurements are shown. The pre-edge peak, which can distinguish between VC_1−*x*_ and VN, is analysed in detail. A new approach for a quantitative comparison with experimental reference spectra is presented, taking into account the self-absorption effects.

#### 3.4.1. V–Al–C–N samples at different *X*   

**Figure 6:**
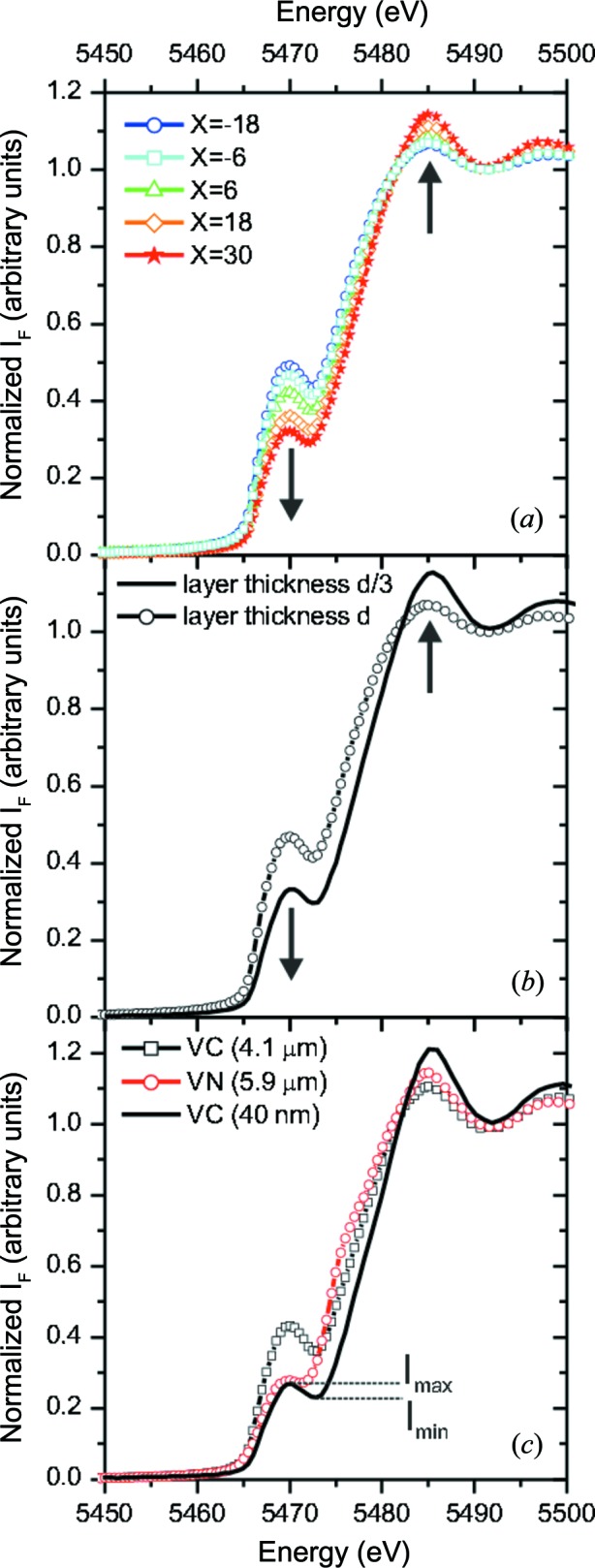
(*a*) XANES region of the vanadium *K* edge for the V–Al–C–N coatings in the range 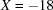
 mm to 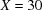
 mm. The pre-edge peak decreases with increasing *X*, while the white line increases as indicated by arrows. (*b*) Influence of the coating thickness on the characteristic edge features at 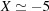
 mm. The measurement of the thick V–Al–C–N coating deposited on cemented carbide is indicated by symbols; the line corresponds to the coating on Si(001) with about 1/3 of the layer thickness. (*c*) XANES spectrum of the 40 nm VC coating (black line), the VC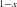 coating (black squares) and the VN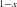 coating (open circles) measured for 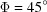.

Fig. 6 ▶(*a*) shows the XANES region of the vanadium *K* edge, measured for the V–Al–C–N coatings in the range *X* = −18 mm to *X* = 30 mm. For all spectra, the pre-edge peak is observed at *E* ≃ 5470 eV, with a subsequent minimum at *E* ≃ 5472.5 eV and the white line at *E* ≃ 5490 eV. The strongest pre-edge peak is observed for *X* = −18 mm. It decreases with increasing *X*, while the white line increases as indicated by black arrows.

The pre-edge peak corresponds to a 1*s* 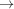 3*d* transition, which is dipole forbidden and quadrupole allowed for V in octahedral sites (*e.g.* in the NaCl structure of VC_1−*x*_ and VN) but dipole allowed for V in tetrahedral sites (Wong *et al.*, 1984 ▶) (*i.e.* in the wurtzite structure expected for V–Al–C–N with high Al content). Therefore, for octahedral sites only a weak pre-edge peak is expected, while for tetrahedral sites a sharp intense pre-edge peak should be found. All observed spectra of V–Al–C–N have the typical shape expected for the NaCl structure.

Fig. 6 ▶(*b*) shows the XANES spectrum of the V–Al–C–N coating deposited on cemented carbide at *X* ≃ −5 mm (open circles), together with the spectrum of a coating deposited on Si(001) with a similar composition but only about one-third of the thickness (solid line). Compared with the thin coating, the spectrum of the thick coating shows an increase of the pre-edge peak and a decrease of the white line. This indicates that self-absorption has a strong influence on the pre-edge peak and cannot be neglected for our samples. In the following, the influence of the chemical composition and self-absorption on the pre-edge peak will be discussed in detail.

#### 3.4.2. Pre-edge peak and chemical composition   

Fig. 6 ▶(*c*) shows the spectra of the 40 nm VC coating (black line), the VC reference coating (black squares) and the VN reference coating (open circles) measured at ϕ = 45°. For both VC reference coatings, a pronounced pre-edge peak is observed, but for VN this peak is reduced to a shoulder. This observation is consistent with reference spectra for VC and VN reported in the literature (Wong *et al.*, 1984 ▶; López-Flores *et al.*, 2008 ▶).

The pre-edge peak-shape difference cannot be explained by self-absorption effects, since the VC pre-edge peak is also clearly visible for the 40 nm coating where self-absorption effects are negligible. It might be related to a slight distortion of the octahedral coordination of V in VC_1−*x*_. In contrast with stoichiometric VN, stoichiometric VC with a perfect octahedral coordination is not stable (Gusev *et al.*, 2001 ▶; Lipatnikov, 2005 ▶), although the changed chemical environment might also contribute to the peak height.

**Figure 7:**
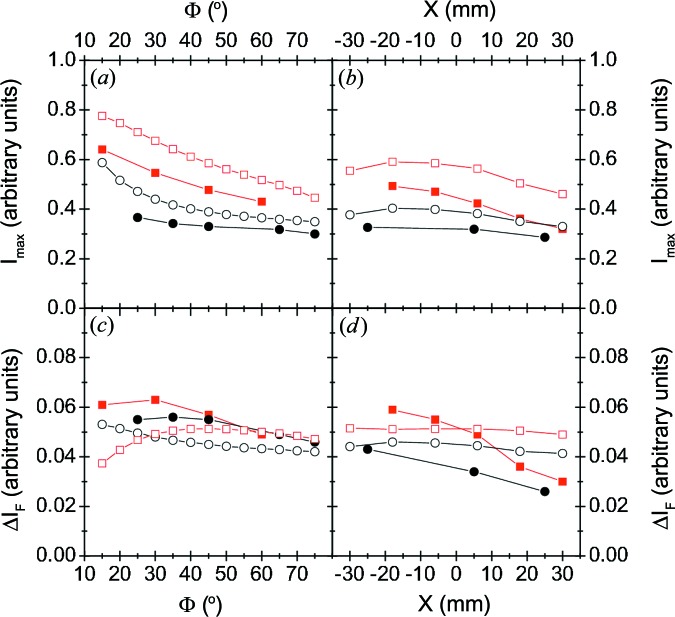
Comparison of the pre-edge peak intensity of the V–Al–C–N coatings and the calculated pre-edge peak intensity of VC with similar coating thickness. (*a*) Maximum intensity *I*_max_ as a function of the incident angle Φ, (*b*) *I*_max_ measured at 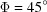 for different positions *X* below the target, (*c*) intensity difference Δ*I*_F_ = *I*_max_ − *I*_min_ as a function of Φ, and (*d*) Δ*I*_F_ at different *X*. Experimental data for coatings on cemented carbide (filled squares) and on Si(001) (black dots) are shown. The calculated values for VC assuming the thickness of the coatings on cemented carbide and on Si(001) are represented by open squares and open circles.

The shape of the pre-edge peak can be described by the maximum of the normalized fluorescence intensity, *I*_max_, the minimum after the pre-edge peak, *I*_min_, and the intensity difference Δ*I*_F_ = *I*_max_ − *I*_min_ (see Fig. 7 ▶ *c*). Δ*I*_F_ ≲ 0.01 for the VN reference coatings, Δ*I*_F_ ≃ 0.07 for the VC reference coating and Δ*I*_F_ ≃ 0.04 for the 40 nm VC reference coating. For the V–Al–C–N coatings, 0.025 ≲ Δ*I*_F_ ≲ 0.06, *i.e.* in between the values of the reference coatings.

For the VC and VN reference coatings, the maximum intensity is measured at *E* = 5470.0 eV. The minimum position depends on the material: for VC, the minimum is at *E* = 5473.0 eV, while for VN it is at the slightly lower value *E* = 5471.5 eV. The reference samples were measured at several incident angles ϕ in the range 15–75°. It was found that the positions of *I*_max_ and *I*_min_ are independent of ϕ, *i.e.* independent of the self-absorption.

For the V–Al–C–N coatings, *I*_max_ is at the same position as for the reference samples, while *I*_min_ shifts from *E* = 5472.5 eV at *X* = −18 mm to *E* = 5472.0 eV at *X* = 30 mm. For small *X*, the minimum position is closer to the value observed for the VC reference sample, while for larger *X* it shifts in the direction of the value observed for the VN reference sample. The observed shift of the minimum suggests that, with increasing N content, the number of V—N bonds increases. This interpretation is supported by the full miscibility of VN and VC reported by Duwez & Odell (2009 ▶).

#### 3.4.3. Influence of the self-absorption effect on the pre-edge peak   

Fig. 7 ▶ summarizes the quantitative analysis of the measured pre-edge peak for the V–Al–C–N coatings on cemented carbide (filled squares) and on Si(001) (black dots). Figs. 7 ▶(*a*) and 7 ▶(*c*) show *I*_max_ and Δ*I*_F_ as a function of the incident angle ϕ, and Figs. 7 ▶(*b*) and 7 ▶(*d*) show *I*_max_ and Δ*I*_F_ as a function of the position *X*. The experimental data are compared with the calculated pre-edge peak of VC, assuming the measured thickness and composition of the V–Al–C–N coatings on cemented carbide (open squares) and on Si(001) (open black circles) for the calculation of the self-absorption effect.

 Even with these simple assumptions, the calculated values reproduce well the angle-dependent values for *I*_max_ and Δ*I*_F_: (1) the decrease in *I*_max_ with increasing ϕ; (2) the offset between the *I*_max_ values for the thin coatings on Si and the thick coatings on cemented carbide; and (3) the decrease in Δ*I*_F_ for ϕ ≳ 30°. For ϕ ≲ 30°, slight deviations are observed. Since the calculations only take into account the characteristic VC spectrum and the self-absorption, the reproduced features are clearly related to the self-absorption effect.

For all V–Al–C–N coatings, the calculated values reproduce well the variation in *I*_max_ as a function of *X* and the offset between the values for the thin coatings on Si and the thick coatings on cemented carbide. These observations are mainly related to the self-absorption. The measured Δ*I*_F_ are in between the values for the VN and VC reference coatings. While the calculated Δ*I*_F_ values (assuming the thickness of the V–Al–C–N coatings) vary only slightly, the measured values decrease significantly with increasing *X*. This is consistent with the assumption that both V—N and V—C bonds contribute to the measured signal and that the V—N contribution increases with increasing *X*.

#### 3.4.4. Summary of XANES results   

For all positions *X*, a weak pre-edge peak related to the NaCl structure was observed. It could be shown that the detailed shape of the pre-edge peak varies with the film thickness and the chemical composition. The influence of the self-absorption on the pre-edge peak was studied in detail for VC, thus allowing the identification of the changes related to the chemical composition. The amplitude changes and the shift of the minimum after the pre-edge peak indicate a coexistence of V—N and V—C bonds. The contribution of V—N increases with increasing N content, as expected for a mixed V–Al–C–N phase.

### 3.5. Extended X-ray absorption fine-structure spectroscopy   

**Figure 8:**
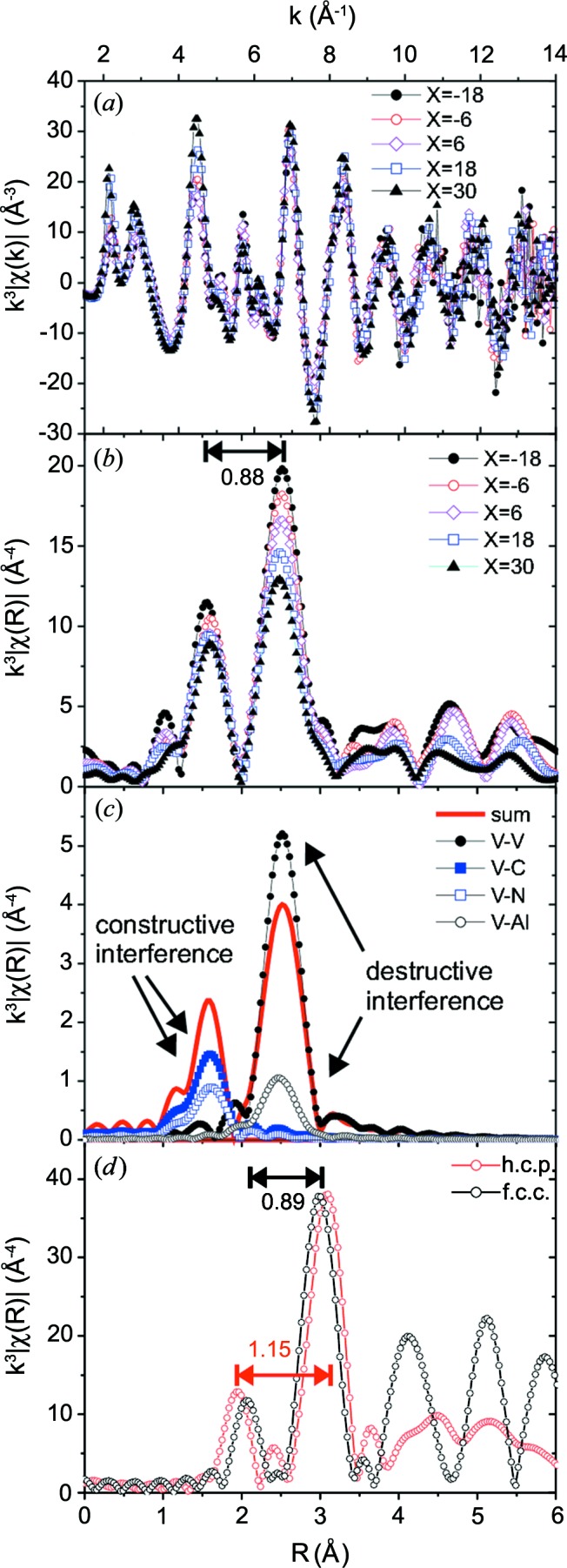
(*a*) Measured EXAFS spectra for different positions *X* below the target shown in *k* space, (*b*) absorption-corrected spectra plotted in *R* space, (*c*) contributions of the different scattering paths to the simulated f.c.c. EXAFS spectrum, and (*d*) comparison of the simulated EXAFS spectra for the f.c.c. and h.c.p. crystal phases.

EXAFS measurements were performed for the V–Al–C–N coatings deposited on cemented carbide in the range *X* = −18 mm to *X* = 30 mm below the target. Fig. 8 ▶(*a*) shows the measured EXAFS spectra weighted by *k* ^3^ in *k* space, and Fig. 8 ▶(*b*) shows the absorption-corrected (but not phase-shift-corrected) EXAFS signal weighted by *k* ^3^ in *R* space. The symbols corresponding to the different *X* are indicated in the legends.

Two peaks in the range *R* = 1–3 Å are clearly visible. For all samples, the distance between the peaks is 0.88 Å. We found that the absorption correction influences the intensities of the peaks but does not change their intensity ratio or shape. The smaller peaks at *R* > 3 Å show that the crystalline order of all the coatings extends beyond the first two shells.

Fig. 8 ▶(*d*) shows the simulated EXAFS spectra for VC in the f.c.c. NaCl structure (black circles) and the h.c.p. wurtzite structure (grey circles; red in the electronic version of the journal). The lattice parameter *a* = 4.163 Å was assumed for cubic VC_1−*x*_ (ICSD-159870 for VC in the NaCl structure; ICSD, 2013 ▶). The hexagonal phase is not stable for VC_1−*x*_, so the lattice parameters of AlN (*a* = 3.112, *c* = 4.981 Å) were used (ICSD-54697 for hexagonal AlN; ICSD, 2013 ▶).

For the cubic structure, the distance between the peaks is 0.89 Å, while for the h.c.p. structure the distance of 1.15 Å is much larger. This difference does not depend significantly on small variations of the lattice parameters, *e.g.* due to chemical composition. Therefore, it can be used to distinguish between the h.c.p. and f.c.c. structures, as has been shown by Tuilier *et al.* (2007 ▶) for hexagonal and cubic TiAlN. In our case, the measured distance is nearly identical to the distance expected for cubic VC, indicating that the V atoms are mainly incorporated into the f.c.c. structure.

The spectra measured at different *X* are very similar (Fig. 8 ▶ *b*), and only the peak intensity ratio between the second and first peaks changes. The peak ratio can be influenced by the occupancy of the shells and by the Debye–Waller factor σ, which describes the thermal and positional disorder of a mixed system. However, in the case of V–Al–C–N, the peak ratio is also influenced by the composition. As an example, Fig. 8 ▶(*c*) shows the simulated EXAFS spectrum for f.c.c. V–Al–C–N with four C atoms and two N atoms in the first shell, and nine V atoms and three Al atoms in the second shell. Both the first (nonmetal) shell and the second (metal) shell are fully occupied, with *N*(C + N) = 6 atoms and *N*(V + Al) = 12 atoms, respectively. The contributions of the single scattering paths V—N and V—C have the same phase and add up constructively. Since the scattering of the two atom types is very similar, they influence the first-shell EXAFS peak in a similar way. This makes it very difficult to distinguish C and N from the EXAFS signal. The contributions of the single scattering paths V—V and V—Al are phase shifted by π. The two paths add up destructively, *i.e.* the peak height decreases with increasing Al content. Similar observations were reported by Tuilier *et al.* (2007 ▶) for TiAlN. This similarity between the phase shifts of Ti and V is expected, since the elements are immediate neighbours in the periodic table.

For our samples, the peak ratio between the second and first peaks decreases with increasing *X*, *i.e.* with increasing Al content. This is expected for a mixed V–Al–C–N phase, because of the destructive interference between V and Al. Therefore, the data were fitted using a mixed f.c.c. phase, with Al or V on the metal site and *N*(V + Al) = 12 atoms. Since C and N cannot be reliably distinguished, the nonmetal sites were assumed to be occupied by C. The occupancy *N*(C) was varied, since VC_1−*x*_ can form many similar nonstoichiometric cubic phases with different carbon content.

All presented fits were performed in the range *R* = 1–3 Å with *E*_0_ = 5 eV. In the NaCl structure of VC, only single scattering paths exist with *R* < 3 Å. These paths were taken into account. The first multiple scattering path that might give a small contribution to the second peak is found at *R* = 3.5 Å. However, the structural disorder of a mixed system reduces significantly the contributions of multiple scattering paths. Therefore, the paths used for the fit are the V—C path with *R*_V—C_ = 2.082 Å, the V—(V,Al) path with *R*_V—(V,Al)_ = 2.944 Å and the third-neighbour V—C path with *R*_V—C2_ = 4.163 Å. As an example, the path lengths were calculated assuming the cubic VC unit cell. V—C2 is outside the fitting range but contributes to the second peak. If it is not included in the fit model, *N*(C) is underestimated by two to three atoms.

**Figure 9:**
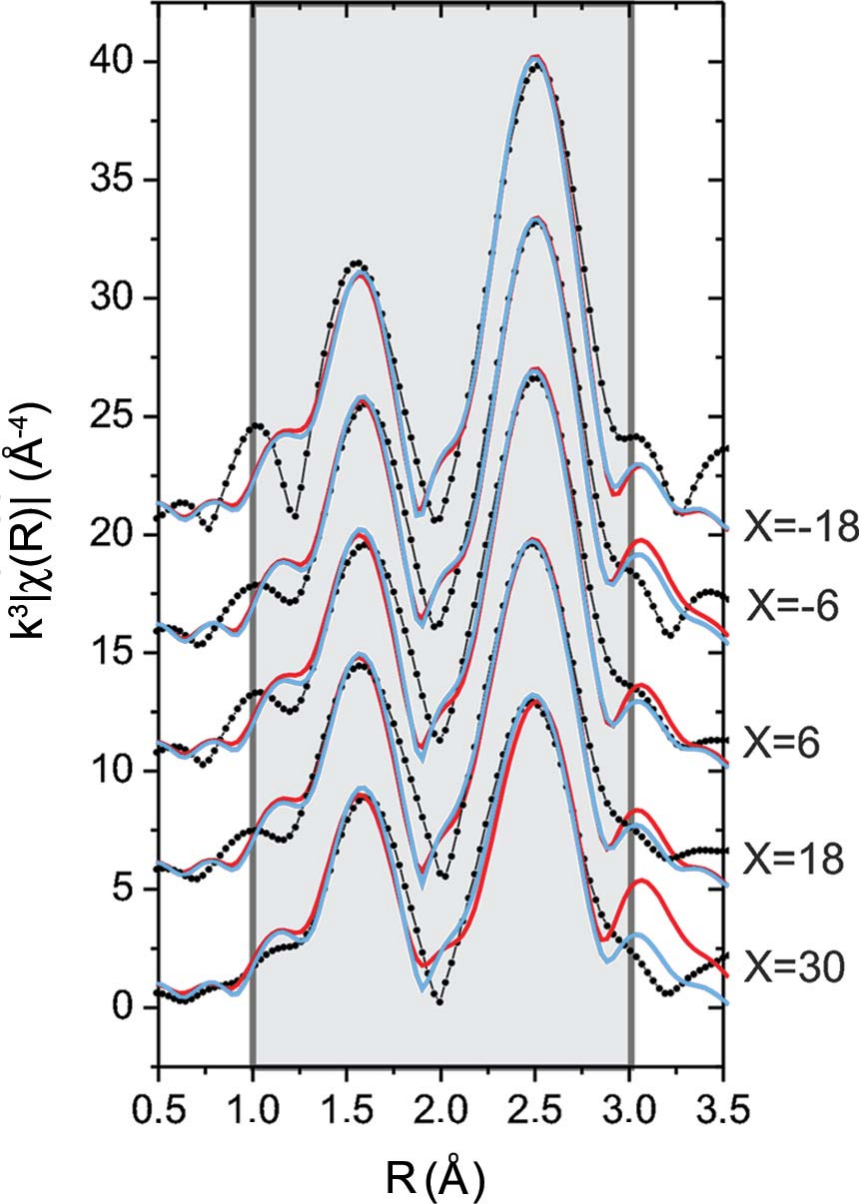
Position-dependent Fourier-transformed EXAFS signal (symbols), fit with the maximum possible Al content giving a good fit result (mid-grey line, or red in the electronic verson) and fit assuming no Al in the structure (pale grey or blue line). The grey background indicates the *R* range used for the fit.

All measured EXAFS spectra were fitted using the model of a mixed crystal, assuming various Al contents. Owing to the destructive interference between Al and V, nearly identical fit curves can be created by changing *N*(Al) and *N*(C). Increasing *N*(C) reduces the peak ratio, though this can be compensated by decreasing *N*(Al). Fig. 9 ▶ shows the best fits for the coatings at different *X*, assuming the maximum number of Al atoms giving a good fit result (mid-grey or red line). The *R* factor of the fit is typically in the range 0.25–0.4. The grey area indicates the *R* range used for the fit. The fit parameters are summarized in Table 1 ▶ (upper part).

The fit results are consistent with the expectations for a mixed phase: *R*_V—(V,Al)_ and *R*_V—C_ decrease with increasing *X*, as expected for a mixed crystal with increasing Al content. *N*(C) is close to the nominal value for the ideal f.c.c. structure, *N*(C) = 6. The Al content increases with increasing *X*. Furthermore, the ratio Al/(Al + V) determined from the EXAFS fit is similar to the value calculated from the chemical composition of the coatings measured by EPMA.

The fitted *R*_V—(V,Al)_ values for the V–Al–C–N coatings and the VC reference coating (not shown here) are about 0.05 Å smaller than the reference values calculated from the VC unit cell. This discrepancy cannot be explained by concentration changes. Such large offsets due to the calculated phase shift were reported in the literature for Pt–O (Koningsberger *et al.*, 2000 ▶).

The amplitude reduction factor 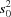, which takes multi-electron effects into account, is in the range 0.8–1, which compares well with the typically expected values (Li *et al.*, 1995 ▶). The Debye–Waller factors 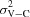 and 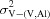 are both approximately 0.005 Å^2^ for all samples, indicating that the structural disorder does not change significantly with increasing Al content. This is consistent with the XRD results.

In order to demonstrate the influence of the destructive interference between V and Al on the fit result, Fig. 9 ▶ also shows fit results assuming *N*(Al) = 0 (pale grey or blue line), *i.e.* assuming the extreme case that all V atoms are incorporated in a VC_1−*x*_ phase. The fit parameters are summarized in Table 1 ▶ (lower part).

The fit results are very similar to the fit with maximum Al content. *R*_V—V_ decreases, as found already for the model of a mixed crystal, while *R*_V—C_ increases slightly, but the decrease is only small compared with the error bar. The main result of the VC_1−*x*_ model is that, independent of the sample, a good fit is only possible for *N*(C) >> 6. This might be reasonable assuming very small crystals, since for small crystals with a size of around 1–2 nm the contribution of the surface atoms plays a significant role. However, this model is not consistent with the fact that both the XRD and the EXAFS results indicate larger well ordered crystallites. Additionally, the XPS measurements show a significant contribution of C—C bonds. Taking into account the chemical composition measured by EPMA, this contradicts *N*(C) >> 6. Furthermore, the 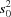 values are very low compared with the typical range of 0.7–1 (Li *et al.*, 1995 ▶), which makes this fit model extremely unlikely.

Summarizing the EXAFS results, all spectra show the characteristic features of a vanadium-containing f.c.c. structure. The spectra could be fitted with two models: cubic mixed V–Al–C–N and cubic V—C. Only the fit assuming cubic mixed V–Al–C–N leads to reasonable values for all parameters. The result of this model is a mixed f.c.c. V–Al–C–N phase with the expected chemical composition, *i.e.* increasing Al content with increasing sample position *X* below the target. The size of the unit cell decreases with increasing Al content, as expected from Vegard’s law.

## 4. Summary   

The short-range order, long-range order and chemical bonds of simultaneously deposited V–Al–C–N coatings with a composition gradient along the *X* axis were studied.

The XRD and XAS measurements confirm the cubic NaCl structure for the studied concentration range. No indication of other crystalline phases was found.

According to Vegard’s law, for a mixed phase with increasing AlN content a decreasing size of the unit cell is expected. This expected decrease was found for the relaxed unit cell determined by XRD and for the atomic distances determined from the EXAFS measurements. However, the decrease might also be due to a mixture of VC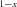 and VN (Duwez & Odell, 2009 ▶), and even a certain number of unoccupied nonmetal sites might lead to a decrease of the unit cell (Lipatnikov, 2005 ▶). Since for cubic VC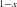 and AlN a wide range of lattice parameters is reported, the decrease of the unit cell alone is not sufficient to conclude on a mixed (V,Al)(C,N) phase.

The XPS measurements show that the V–Al–C–N coatings contain both nitrides and carbides, but Al—C and Al—N, as well as V—C and V—N, cannot be distinguished. For all samples, the XPS measurements confirm the coexistence of a phase containing C—C bonds. The XANES signal at the V *K* edge is sensitive to the difference between V—C and V—N. Both VC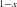 and VN have a cubic unit cell, but the pre-edge peak shape is much less pronounced for VN. For our coatings, the pre-edge peak depends also on self-absorption effects. This was demonstrated experimentally by measuring coatings with different thickness but similar composition, and by calculating the expected self-absorption effect for VC. For the V–Al–C–N coatings, the self-absorption effect is mainly related to the coating thickness and is less influenced by the composition. Even taking into account the distortion due to the self-absorption effect, the XANES measurements indicate that both V—N and V—C bonds contribute to the pre-edge peak.

The EXAFS signal is sensitive to the mixture of V and Al. It was shown that the EXAFS signal is not unique for f.c.c. (V,Al)(C,N) with different compositions. Similar fit curves can be produced with different nearest neighbour occupancies. By combining the EXAFS fit result with the long-range order observed by XRD and the chemical composition determined by EPMA, it was found that only a cubic (V,Al)(C,N) mixed phase is consistent with all results.

In summary, the here studied V–Al–C–N samples deposited by reactive RF magnetron sputtering consist of a cubic (V,Al)(C,N) mixed phase and a coexisting C—C-containing phase, which might be a grain boundary phase, small carbon clusters or an a-C:N phase. The observed coexisting phases are expected to be favourable for hard coating applications. The presented results are very similar to the observations for Ti–Al–C–N, with a comparable TM/(TM + Al) ratio but different C/(C + N) ratio (Shieh & Hon, 2002 ▶; Lackner *et al.*, 2004 ▶; Stueber *et al.*, 2005 ▶; Nose *et al.*, 2010). This supports the assumption that many experimental results of the well known system Ti–Al–C–N can be transferred to the new material system V–Al–C–N. Similar to Ti–Al–C–N, the experimentally observed cubic (V,Al)(C,N) phase is the predicted thermodynamically stable phase for V–Al–C–N coatings with 0.1 < TM/(TM + Al) < 0.4. The transition from metastable f.c.c. to metastable h.c.p. mixed phases should occur at much higher Al content. Therefore, it might be possible to increase the hardness of the material further by increasing the AlN content but maintaining the f.c.c. structure. In future, the study of V–Al–C–N will be extended to this concentration range.

## Figures and Tables

**Table 1:** Parameters of the EXAFS fit The upper fit parameters result from the model of a mixed crystal with maximum Al content; for the lower fit parameters a VC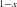 phase was assumed. Values in parentheses are uncertainties on the least significant digit.

*X* (mm)	18	6	6	18	30
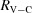 ()	2.026 (9)	2.025 (8)	2.025 (9)	2.025 (10)	2.023 (18)
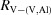 ()	2.891 (7)	2.891 (6)	2.887 (8)	2.874 (10)	2.879 (17)
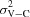 (^2^)	0.006 (3)	0.004 (2)	0.006 (6)	0.005 (3)	0.004 (5)
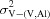 (^2^)	0.006 (1)	0.006 (1)	0.006 (1)	0.006 (2)	0.006 (3)
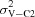 (^2^)	0.011 (9)	0.002 (6)	0.005 (6)	0.006 (10)	0.000 (11)
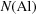	2.0 (5)	2.0 (5)	3.0 (5)	3.0 ( 5)	4.0 (5)
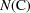	7.0 (10)	7.0 (10)	6.5 (10)	7.5 (10)	5.5 (10)
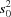	0.96 (10)	0.82 (7)	0.96 (11)	0.79 (11)	0.88 (20)

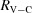 ()	2.031 (10)	2.034 (8)	2.035 (8)	2.034 (8)	2.037 (10)
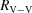 ()	2.890 (7)	2.890 (6)	2.886 (7)	2.872 (7)	2.876 (9)
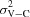 (^2^)	0.005 (3)	0.004 (2)	0.005 (5)	0.005 (3)	0.003 (3)
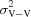 (^2^)	0.006 (1)	0.005 (1)	0.006 (1)	0.005 (1)	0.005 (2)
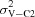 (^2^)	0.011 (10)	0.004 (6)	0.008 (9)	0.009 (8)	0.005 (10)
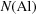	0	0	0	0	0
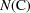	9.5 (10)	9.5 (10)	10.5 (10)	12.0 (10)	10.5 (10)
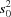	0.67 (7)	0.58 (5)	0.55 (5)	0.46 (5)	0.41 (5)
